# Clinical investigation of hypoxia-inducible factors: getting there

**DOI:** 10.1172/JCI176253

**Published:** 2024-02-01

**Authors:** Gregg L. Semenza

**Affiliations:** Armstrong Oxygen Biology Research Center, Institute for Cell Engineering, and Department of Genetic Medicine, Johns Hopkins University School of Medicine, Baltimore, Maryland, USA.

As part of the *JCI*’s 100th-anniversary celebration, I reflect here on a few of the many papers published in *JCI* that have advanced our understanding of the physiological and pathological responses to hypoxia, with an emphasis on the hypoxia-inducible factors (HIFs), which mediate transcriptional responses to decreased O_2_ availability. HIFs consist of an O_2_-labile HIF-1α, HIF-2α, or HIF-3α subunit and a constitutively expressed HIF-1β (also known as ARNT) subunit. The HIF prolyl hydroxylases use O_2_ and α-ketoglutarate as substrates to modify the HIF-α subunits by inserting an oxygen atom into a prolyl residue (either P402 or P564 in human HIF-1α) under normoxic conditions; the hydroxylated proteins are selectively bound by the von Hippel–Lindau tumor suppressor protein (VHL), which recruits a ubiquitin-protein ligase that ubiquitinates the HIF-α subunits, thereby marking them for proteasomal degradation (1). In contrast, under hypoxic conditions, the hydroxylases are inhibited and HIF-α subunits rapidly accumulate, providing a direct mechanism to transduce changes in O_2_ availability to changes in HIF activity (1). I also briefly highlight the role of HIFs in erythropoiesis, pulmonary vascular biology, angiogenesis, metabolism, cancer, and diabetic eye diseases.

In 1999, my group reported that mice, which were heterozygous for a knockout allele at the locus encoding HIF-1α, manifested impaired responses to chronic hypoxia (exposure to a 10% O_2_ ambient environment for 3 weeks) (2). The reason for studying heterozygotes was that the homozygotes, which completely lacked HIF-1α expression, died at midgestation, with defects in heart development, tissue vascularization, and red blood cell production, thereby demonstrating that HIF-1α was required for development of all three components of the circulatory system. Analysis of the heterozygotes provided a connection between the role of HIF-1 in development and its role in physiology. Mice exposed to chronic hypoxia develop erythrocytosis and pulmonary hypertension, and these responses were impaired in the heterozygotes. The effect on erythrocytosis remains unexplained; HIF-2 is now considered the primary regulator of adult erythropoiesis through its regulation of EPO production, based on studies of conditional knockout mice (3) and studies demonstrating that missense mutations in the HIF pathway that increase HIF-2α expression result in the phenotypic triad of hereditary erythrocytosis, pulmonary hypertension, and thrombosis in humans and mice (4).

Hypoxic pulmonary vasoconstriction associated with lobar pneumonia is an adaptive response to hypoxia, as it shunts blood away from areas of lung that are not ventilated, whereas panlobar vasoconstriction is a maladaptive response to ambient hypoxia, as it leads to decreased blood oxygenation. Mice heterozygous for a HIF-2α–knockout allele were also protected from the development of pulmonary hypertension (5). The roles of HIF-1 and HIF-2 signaling in the pathogenesis of pulmonary hypertension are broad and essential, with complex feed-forward circuits, both within and between, pulmonary artery endothelial and smooth muscle cells, as delineated in an excellent review (6), which also discussed pharmacologic targeting of HIFs as a potential therapy in this disorder. The critical role of HIF-1 in the vascular response of lung transplants to chronic rejection was also established by a *JCI* paper; data from a mouse model suggested that adenoviral stimulation of HIF activity could prevent lung transplant rejection by maintaining integrity of the microvasculature and tissue perfusion during chronic rejection (7).

Hypoxia induces the HIF-dependent expression of angiogenic growth factors, such as VEGFA, which stimulate new blood vessel formation, thereby increasing tissue perfusion and oxygenation (8). One of the early studies demonstrating that VEGFA administration could augment ischemia-induced vascularization was published in *JCI* (9). Unfortunately, the study utilized young and previously healthy mice, which are not a suitable model for ischemic cardiovascular disease, which is a disease associated with aging. It took the field a long time to learn this lesson.

Two of the most critical adaptations to localized tissue hypoxia are the increased production of angiogenic growth factors to stimulate new blood vessel formation as a means to increase O_2_ delivery and the switch from oxidative to glycolytic metabolism as a means to decrease O_2_ consumption (10). The co-option of these physiological responses by cancer cells is best illustrated by the clear cell renal cell carcinoma (ccRCC) in patients with the von Hippel–Lindau syndrome, in which one copy of the *VHL* gene is inactivated by germline mutation and the other by somatic mutation within kidney cells. It is a peculiarity of this particular cancer that during tumor progression many ccRCCs lose HIF-1α expression and are driven solely by dysregulated HIF-2α expression; this led to the development of belzutifan, a drug that selectively binds to HIF-2α and blocks its dimerization with HIF-1β, thereby causing loss of HIF-2 transcriptional activity and a dramatic therapeutic benefit in patients with advanced ccRCC whose prognosis was previously bleak (11).

Among its many effects in breast cancer, HIF-1 was recently shown to control the production of small extracellular vesicles, which were shown to drive cancer progression via multiple mechanisms (12).

Another major recent advance has been the discovery that HIFs play critical roles in mediating the ability of cancer cells of many types, including breast, colorectal, and liver cancer, to evade killing by the immune system and that small-molecule inhibitors of HIF-1 and HIF-2 dramatically improve the response to immune checkpoint inhibitors (anti-CTLA4, anti–PD-1 or anti–PD-L1 antibody) in mouse models (13, 14). These HIF inhibitors have been remarkably well tolerated in mouse models, with no changes in mouse appearance, behavior, or body weight, suggesting that a therapeutic window exists for HIF inhibition, but, of course, this issue can only be definitively resolved by clinical trials.

Diabetic eye disease is a major and increasing cause of progressive vision loss and blindness in the adult US population. Both HIF-1 and HIF-2 play major roles in retinal neovascularization, and, in mouse models, a small-molecule dual HIF-1/HIF-2 inhibitor safely and effectively blocks the development of macular edema and ischemic retinal neovascularization, which are the causes of vision loss associated with diabetes (15). These studies have highlighted that a large battery of angiogenesis-associated gene products are expressed in a HIF-dependent manner in diabetic eye diseases and that, whereas current therapies target only one of them (VEGFA), HIF inhibitors have a much broader effect on gene expression, which may translate into a higher response rate among patients with diabetic eye diseases, less than half of whom respond well to anti-VEGFA therapies (15). Because drugs for these conditions are delivered by intraocular injection, systemic adverse effects are not a major consideration.

The last quarter of the *JCI*’s first century has seen dramatic advances in our understanding of the role of HIFs in the pathogenesis of diseases, with major effects on morbidity and mortality. Looking forward, I anticipate that further pharmacologic targeting of HIFs will provide therapeutic benefit in several of these disorders. Table 1 lists the approved and some of the potential therapeutic applications for pharmacologic HIF inducers and HIF inhibitors. Let’s hope to see more patients replace mice as the recipients of novel therapies in the *JCI*. Watch this space.

## Figures and Tables

**Table 1:**
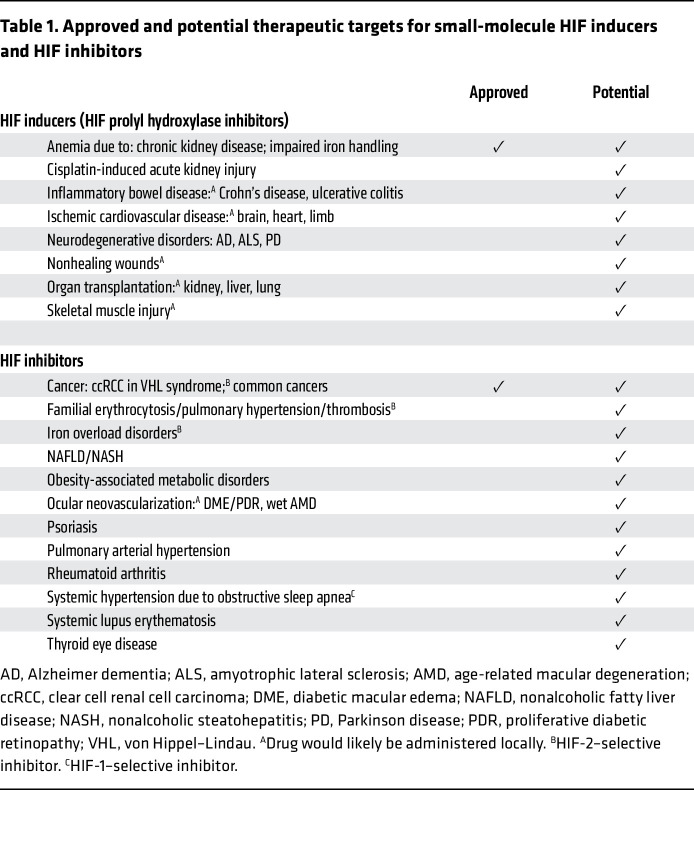
Approved and potential therapeutic targets for small-molecule HIF inducers and HIF inhibitors

## Version 1 02/01/2024

Electronic publication
